# Aripiprazole use and lowering the risk of breast cancer in patient with schizophrenia in a national cohort study

**DOI:** 10.1371/journal.pone.0324257

**Published:** 2025-06-04

**Authors:** Po-Hsun Hou, Gung-Ruei Chang, Ying-Ting Chao, Yu-Teng Chang, Da-Han Hou, Shiau-Shian Huang

**Affiliations:** 1 Department of Psychiatry, Taichung Veterans General Hospital, Taichung, Taiwan; 2 Department of Post-Baccalaureate Medicine, College of Medicine, National Chung Hsing University, Taichung, Taiwan; 3 Department of Veterinary Medicine, National Chiayi University, Chiayi, Taiwan; 4 Department of Public Health, Institute of Epidemiology and Preventive Medicine, College of Public Health, National Taiwan University, Taipei, Taiwan; 5 Department of Medical Research, National Taiwan University, Taipei, Taiwan; 6 National Chung Hsing University, Institude of Biomedical Science, Taichung, Taiwan; 7 Department of Chinese Medicine, China Medical University, Taichung, Taiwan; 8 College of Medicine, National Yang Ming Chiao Tung University, Taipei, Taiwan, Repubic of China; 9 School of Public Health, National Defense Medical Center, Taipei, Taiwan; 10 Department of Medical Education, Taipei Veterans General Hospital, Taipei, Taiwan, Repubic of China; 11 Nankung psychiatric Hospital, Keelung, Taiwan; 12 Bali Psychiatric Center, Ministry of Health and Welfare, New Taipei, Taiwan; Chiba Daigaku, JAPAN

## Introduction

Breast cancer stands as the most prevalent cancer among women globally, boasting a lifetime prevalence rate of approximately 12% [1]. Established risk factors for breast cancer include obesity [2], diabetes [3], and lower number parity, which may increase the risk of breast cancer, luminal subtype [4,5]. Some studies also suggest that parity is not associated with tumor size, histological grade, or the proportion of breast cancer subtypes [6]. Notably, women with schizophrenia often present with an elevated prevalence of these risk factors [4]. However, epidemiologic data regarding breast cancer prevalence are somewhat conflicting. Some studies have found a heightened incidence of breast cancer in individuals diagnosed with schizophrenia [7,8]. Compounding this concern, studies indicate that women with schizophrenia are less likely to undergo routine breast cancer screening, potentially leading to an underestimation of breast cancer prevalence in this population.

The utilization of antipsychotic medications represents another critical consideration in elucidating the elevated breast cancer risk among women with schizophrenia. Breast cancer is recognized as a hormone-dependent malignancy, with increased prolactin concentrations posing a risk factor [9,10].The neurophysiological regulation of prolactin has been found to be controlled by dopamine, and it is also believed to potentially promote breast cancer cell proliferation [11,12]. Antipsychotics, through their antagonism of dopamine D2 receptors, can elevate prolactin levels [13,14].While a definitive causal relationship between antipsychotic use and breast cancer remains elusive, epidemiological studies suggest that prolonged exposure to antipsychotics, particularly those with prolactin-elevating properties, may elevate breast cancer risk [15].

Aripiprazole, a second-generation antipsychotic, serves as a cornerstone in the treatment of schizophrenia and bipolar disorder [16]. Its pharmacological profile includes serotonin 5-HT1A partial agonist, dopamine D2 partial agonist, and 5-HT2A antagonism. Notably, aripiprazole’s interaction with the D2 receptor is contingent upon dopamine levels, exhibiting antagonist effects under hyperdopaminergic conditions and agonist effects under hypodopaminergic conditions [17,18]. Aripiprazole has demonstrated efficacy in reducing prolactin levels and is recommended in clinical guidelines for managing antipsychotic-induced hyperprolactinemia [19].

A Taiwanese cohort study utilizing Taiwan Insurance Claims Data revealed a heightened risk of breast cancer in female schizophrenia patients compared to the non-schizophrenic cohort [20]. Patients prescribed risperidone, paliperidone, and amisulpride, which possess prolactin-elevating properties, exhibited a 1.96-fold increased risk of breast cancer compared to the non-schizophrenic cohort [20]. A recent Finnish study further supported these findings, indicating that prolonged exposure to prolactin-increasing antipsychotics may augment breast cancer risk in female schizophrenia patients [21]. Given aripiprazole’s prolactin-reducing properties and its clinical utility in managing antipsychotic-induced hyperprolactinemia, our study aims to investigate whether the risk of breast cancer differs in schizophrenia patients treated with aripiprazole compared to those treated with other antipsychotics.

## Methods

### Data source and study population

**Fig 1:**
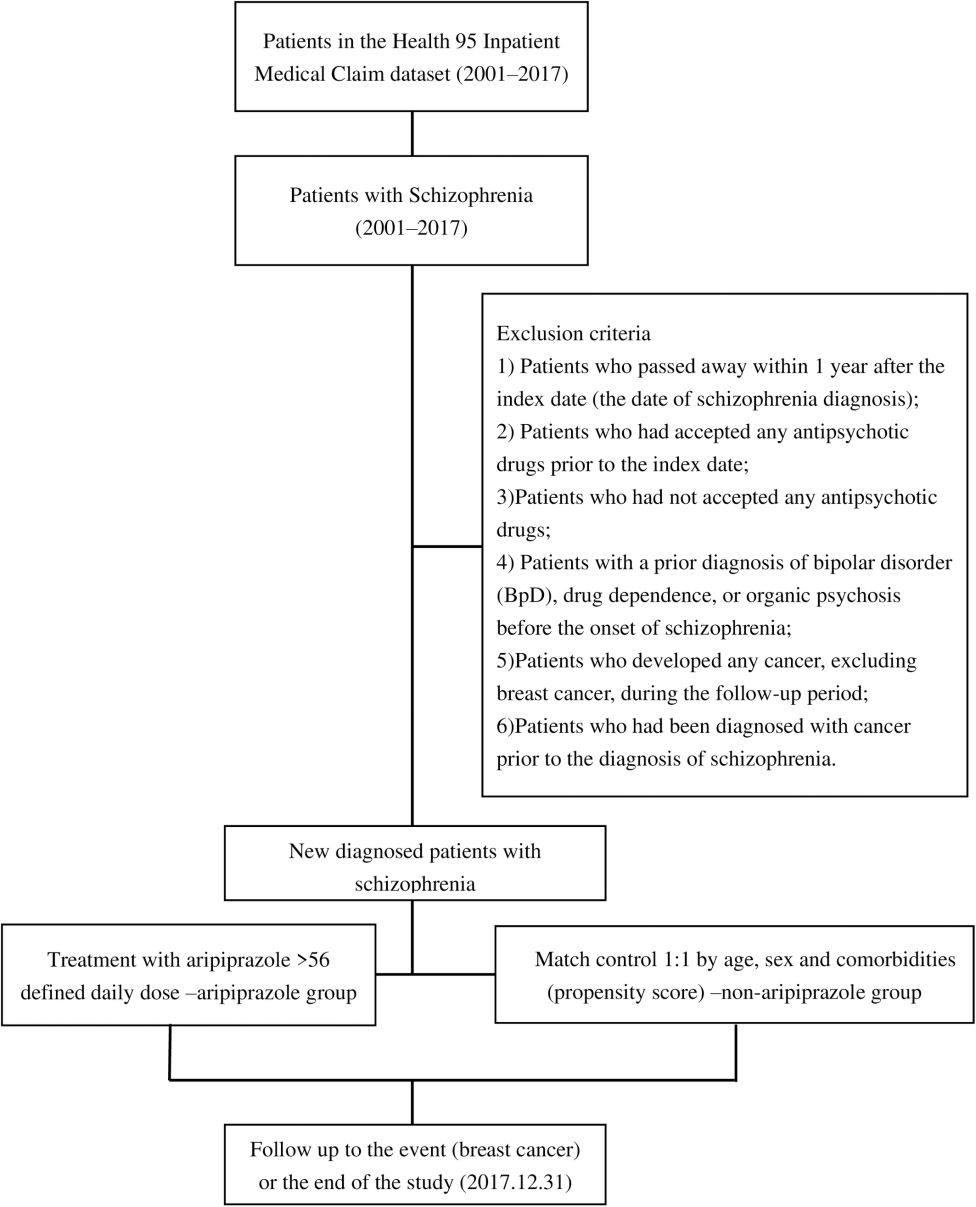
Flowchart illustrating the selection process of study subjects from National Health Insurance Research Database (NHIRD).

In 1995, Taiwan initiated the National Health Insurance program to provide healthcare coverage for all residents, encompassing approximately 97% of the population. The National Health Insurance research database contains the Psychiatric Health Database (PHD), which includes all patients ever diagnosed with any psychiatric disorders (n = 187,117). The dataset comprises basic demographic information and comprehensive medical records (e.g., medication, outpatient services, emergency visits, and hospitalization records).After deleting incomplete data in the Registry for Beneficiaries, newly diagnosed patients with schizophrenia by psychiatrists from 2000 to 2017 were included in the analysis (ICD-9-CM code 295) (Fig 1). Patients were excluded from the study based on the following criteria: 1) Patients who passed away within 1 year after the index date (the date of schizophrenia diagnosis); 2) Patients who had accepted any antipsychotic drugs prior to the index date; 3)Patients who had not accepted any antipsychotic drugs; 4) Patients with a prior diagnosis of bipolar disorder (BpD), drug dependence, or organic psychosis before the onset of schizophrenia; 5) Patients who developed any cancer, excluding breast cancer, during the follow-up period; 6) Patients who had been diagnosed with cancer prior to the diagnosis of schizophrenia.

This study was approved by the Institutional Review Board Committee, Bali Psychiatric Centor, Taiwan M.O.H.W. (IRB serial number: 1110613–01). All data in this study were encrypted and were fully anonymous during data analysis.

### Study group, propensity score, and greedy match

Among the schizophrenia cohort, individuals had previously received aripiprazole treatment, (>56 defined daily dose) constituting the aripiprazole treatment group. Previous studies have shown that antipsychotic medications may require 2–4 weeks to reach a stable concentration in the human body and to exert their effects, with stable treatment requiring more than 4 weeks [22]. Among the schizophrenia cohort, individuals had previously received aripiprazole treatment, (>56 defined daily dose) constituting the aripiprazole treatment group which means patients in study group were treated with adequate dose of aripiprazole. Demographic and clinical characteristics, including age, gender, and comorbidities (both physical and psychiatric), were assessed within this group. Physical comorbidities included diabetes, hypertension, hyperlipidemia, cardiovascular disease, chronic obstructive pulmonary disease, liver disease, renal disease, osteoporosis, and thyroid dysfunction, while psychiatric comorbidities comprised anxiety disorders, substance use disorder, and alcohol use disorder. Patients who had never used aripiprazole formed the non-aripiprazole treatment group. Matching criteria such as diagnosis date, comorbidity propensity scores, age, and gender were employed to select a control group mirroring the characteristics of the study group, utilizing a 1:1 greedy matching method. Subsequently, medical records for both groups were scrutinized for physical and psychiatric comorbidities throughout the study duration.

### Independent variables, covariates, and dependent variable (Event)

Demographic and clinical data were extracted from the PHD database. Demographic variables encompassed gender, urbanization level, and estimated income. Urbanization levels were categorized into seven tiers, with Level I representing the highest degree of urbanization and Level VII the lowest [23]. Clinical information included obesity, pregnancy history, psychiatric and physical comorbidities, medication treatment records, and diagnoses of side effects. Medication investigations involved average dosages of antidepressants, lithium/mood stabilizers, antipsychotic drugs, benzodiazepines, and anticholinergic medications, standardized to defined daily doses (DDD) per the World Health Organization’s guidelines. The DDD is the assumed average maintenance dose per day for a drug used for its main indication in adults [24]. We calculated dose equivalents using the DDD method established by the World Health Organization’s Collaborative Center for Drug Statistics Methodology. This method is widely used for standardizing drug dosages, particularly for central nervous system (CNS) medications such as antipsychotics [24]. Patients’ medication records were obtained from Taiwan’s National Health Insurance Research Database. By applying the DDD methodology, we were able to quantify and compare medication exposure between groups.

The occurrence of breast cancer diagnosis constituted the event of interest, with the date of the first hospital visit for breast cancer defined as the event date (ICD-9 codes: 174.0–174.9; 175.0 or 175.9). Patients were followed from the index date until the diagnosis of breast cancer or until December 31, 2017, with person-days of follow-up calculated accordingly.

### Statistical analysis

Differences between the aripiprazole and non-aripiprazole groups were assessed using the chi-squared test for categorical variables and the t-test for continuous variables, with Bonferroni correction for multiple comparisons. Multiple Cox regression models were employed to examine differences in demographic variables and clinical features between the two groups, adjusting for potential confounders. Covariates included age, sex, physical and psychiatric comorbidities, diagnosis conversion, age of schizophrenia onset, psychiatric medication dosages, electroconvulsive therapy history, and extrapyramidal syndromes. Adjusted hazard ratios (aHRs) with 95% confidence intervals were reported for regression analyses. Statistical analyses were conducted using SAS version 9.4.

## Results

A total of 6,138 schizophrenia patients treated with aripiprazole were identified, along with 6,138 matched controls from the dataset. Table 1 presents the distribution of demographic and clinical characteristics between the aripiprazole treatment group and the non-aripiprazole treatment group. Both cohorts exhibited a predominance of female patients, with comparable distributions (59.30% vs. 58.90%, respectively), while a higher proportion of patients in the aripiprazole treatment group resided in more urbanized areas (79.37% vs 73.92%, p < 0.0001) and has higher income (18.33% vs 16.78%, p < 0.05).

**Table 1:** Demographic and clinical characteristics between the non-aripiprazole group and aripiprazole group patients.

(N = 12,276)	non-aripiprazole group (6,138) 50%		aripiprazole group (N = 6,138) 50%		P-value^2^
Variable	N	%	N	%	
Female	3,615	58.90%	3,640	59.30%	0.646
Urbanization level residence****^1^					<0.0001
I–III	4,537	73.92%	4,872	79.37%	
IV–VII	1,601	26.08%	1,266	20.63%	
Estimated income via insurance (no income)*	1,030	16.78%	1,125	18.33%	<0.05
Physical comorbidity					
Diabetes mellitus*	3,243	52.83%	3,129	50.98%	<0.05
Hypertension**	588	9.58%	695	11.32%	<0.01
Hyperlipidemia****	291	4.74%	493	8.03%	<0.0001
Cardiovascular disease*	455	7.41%	525	8.55%	<0.05
Chronic obstructive pulmonary disease	1,428	23.26%	1,397	22.76%	0.506
Liver disease****	1,156	18.83%	1,346	21.93%	<0.0001
Renal disease	328	5.34%	366	5.96%	0.148
Osteoporosis**	537	8.75%	644	10.49%	<0.01
Thyroid dysfunction**	215	3.50%	272	4.42%	<0.01
Psychiatric comorbidity					
Substance use disorders****	594	9.68%	885	14.42%	<0.0001
Anxiety disorders****	2,730	44.48%	3,552	57.87%	<0.0001
Alcohol use disorder ****	1,283	20.90%	1,618	26.36%	<0.0001
Obesity*	424	6.91%	499	8.13%	<0.05
Pregnancy****	632	10.30%	449	7.31%	<0.0001

*: P-value<0.05;**:p-value<0.01; ****:p-value<0.0001

Notes:

^1^Seven levels, with one being the most urbanized and seven being the least urbanized

^2^P value threshold< 0.0025 for Bonferroni correction

As delineated in Table 1, patients in the aripiprazole treatment group displayed a heightened prevalence of hypertension (11.32% vs 9.58%, p < 0.01), hyperlipidemia (8.03% vs. 4.74%, p < 0.0001), cardiovascular disease (8.55% vs 7.41%, p < 0.05), liver disease (21.93% vs 18.83%, p < 0.0001), osteoporosis (10.49% vs 8.73%, p < 0.01), thyroid dysfunction (4.42% vs 3.50%, p < 0.01), obesity (8.13% vs.6.91%, p < 0.05), and a lower pregnancy rate (7.31% vs.10.30%, p < 0.05). Conversely, the non-aripiprazole treatment group has lower prevalence of diabetes (50.98% vs 52.83%, p < 0.05). Two groups exhibited similar prevalence of other physical comorbidities, including chronic obstructive pulmonary disease, renal disease. With regard to psychiatric comorbidities, compared to the non-aripiprazole treatment group, the aripiprazole treatment group manifested a higher prevalence of substance use disorder (14.42% vs. 9.68%, p < 0.0001), anxiety disorder (57.87% vs.44.48%, p < 0.0001), and alcohol use disorder (26.36% vs.20.90%, p < 0.0001) (Table 1).Moreover, the age of onset for schizophrenia was younger in the aripiprazole treatment group (32.46 ± 13.27vs 33.51 ± 13.04, p < 0.0001). Notably, patients in the aripiprazole treatment group demonstrated a higher frequency of outpatient visits (106.16 ± 121.52 vs 69.11 ± 103.99, p < 0.0001) and more admissions for schizophrenia (3.19 ± 5.89 vs 1.52 ± 3.52, p < 0.0001)(Table 2).

**Table 2:** Medical records, medication records, and side effects between the non-aripiprazole group and aripiprazole group patients.

(N = 12,276)	non-aripiprazole group (N = 6,138) 50%		aripiprazole group (N = 6,138) 50%		P-value^1^
Variables	Mean	SD	Mean	SD	
Age of onset for SCZ****	33.51	13.04	32.46	13.27	<0.0001
Medical records					
Numbers of outpatient visits for SCZ****	69.11	103.99	106.16	121.52	<0.0001
Numbers of admissions for SCZ****	1.52	3.52	3.19	5.89	<0.0001
Medication records (Cumulative sum of DDD)					
Antipsychotics****	2,186.21	3,513.25	2,660.71	3,247.01	<0.0001
Mood stabilizer	769.21	1,410.24	721.37	1,307.80	0.051
Antidepressants****	665.73	1,308.45	1,000.22	1,825.85	<0.0001
Benzodiazepines					
Anxiolytics**	6,596.72	49,601.50	9,585.81	62,880.74	<0.01
Hypnotics*	39,162	263,900	51,416	363,249	<0.05
Anticholinergics*	851.63	1,320.28	806.39	1028.43	<0.05
Sex hormones****	142.87	167.57	228.41	724.49	<0.0001
Variables	N	%	N	%	
Extrapyramidal symptoms****	822	13.38%	1,688	27.49%	<0.0001
Hyperprolactinemia**	82	1.34%	133	2.16%	<0.01
Electro-Convulsive Therapy****	51	0.82%	228	3.71%	<0.0001

*:p-value<0.05;**:p-value<0.01; ****:p-value<0.0001

Schizophrenia: SCZ, Defined Daily Dose: DDD

Note

^1^P value threshold < 0.004 for Bonferroni correction

Psychotropic medication records revealed that patients in the aripiprazole treatment group had higher cumulative dosages of antipsychotics (2,660.71 ± 3,247.01vs2,186.21 ± 3,513.25, p < 0.0001), antidepressants (1,000.22 ± 1,825.85vs665.73 ± 1,308.45, p < 0.0001), anxiolytics (9,585.81 ± 62,880.74 vs6,596.72 ± 49,601.50, p < 0.01), hypnotics(51,416 ± 363,249vs39,162 ± 263,900, p < 0.05), sex hormone(228.41 ± 724.49 vs142.87 ± 167.57, p < 0.0001), and lower cumulative dosages of anticholinergics(806.39 ± 1028.43 vs851.63 ± 1,320.28, p < 0.05), alongside a greater prevalence rate of developing extrapyramidal symptoms (27.49% vs13.38%, p < 0.0001). Additionally, a higher proportion of patients in the aripiprazole treatment group received electroconvulsive therapy compared to the non-aripiprazole treatment group (3.71%vs 0.82%, p < 0.0001) (Table 2).

After adjusting for obesity and other comorbidities (hyperlipidemia, pregnancy, anxiety disorder, substance use disorder, alcohol use disorder), Cox regression analysis revealed that patients undergoing aripiprazole treatment exhibited a decreased likelihood of developing breast cancer compared to those in the non-aripiprazole treatment group (aHR = 0.25, 95% CI = 0.15-.42, p < 0.0001). Furthermore, after adjusting for multiple covariates, older age (aHR = 1.06, 95% CI = 1.05–1.08, p < 0.0001) and alcohol using disorder (aHR = 1.75, 95% CI = 1.11–2.76, p < 0.05) remained significantly associated with a higher hazard ratio for breast cancer in the Cox regression model (Table 3).

**Table 3:** Crude hazard ratio and adjusted hazard ratio from Cox regression analysis for risk of breast cancer among patients with schizophrenia.

Variable	aHR	95% CI		P-value
Crude Hazard Ratio				
Treated with aripiprazole****	0.27	0.16	0.46	<0.0001
Adjusted Hazard Ratio				
Treated with aripiprazole****	0.25	0.15	0.42	<0.0001
Age****	1.06	1.05	1.08	<0.0001
Sex	0.00	0.00	.	0.98
Hyperlipidemia	0.74	0.00	.	1.00
Obesity	0.94	0.53	1.65	0.817
Pregnancy	0.00	0.00	.	1.00
Hyperprolactinemia	0.00	0.00	.	1.00
Anxiety disorder	1.44	0.92	2.25	0.11
Substance disorder	0.87	0.37	2.04	0.75
Alcohol using disorder*	1.75	1.11	2.76	<0.05

*: P-value<0.05;**:p-value<0.01, ***:p-value<0.001,****:p-value<0.0001

aHR adjusted hazard ratio, CI confidence Interval

## Discussion

To our knowledge, this is the first cohort study exploring the relationship between breast cancer incidence and aripiprazole use, either as monotherapy or in combination with other antipsychotics. Our findings suggest that schizophrenia patients receiving aripiprazole treatment, known for its prolactin-lowering effects, may exhibit a reduced risk of breast cancer development.

Schizophrenia, a chronic psychiatric disorder affecting approximately 0.3%-0.7% of the global population, is associated with excess mortality compared to the general population [25,26].Cardiovascular diseases, metabolic syndrome, and certain cancers are more prevalent among schizophrenia patients [27]. Some studies suggest that breast cancer, the most common type of cancer among women, may be more frequent in female schizophrenia patients than in the general population [28]. However, epidemiologic data regarding cancer prevalence in patient with schizophrenia remain conflicting [29–31]. The study has found that traditional antipsychotic drugs may increase the risk of breast cancer, particularly lobular carcinoma, and this is associated with an increase in prolactin levels [21]. Besides other risk factors, hyperprolactinemia, prevalent in approximately 70% of schizophrenia patients requiring long-term antipsychotic treatment, has been implicated in increasing breast cancer risk [32]. Certain types of breast cancer are hormone-dependent, potentially influenced by estrogen, progesterone, and prolactin, which may contribute to breast carcinogenesis [9].As dopamine is a primary inhibitor of prolactin secretion, antipsychotic drugs with D2 receptor antagonism can lead to hyperprolactinemia via blockade of the tuberoinfundibular dopaminergic pathway [33]. Elevated prolactin levels have been linked to increased breast cancer risk, with evidence suggesting a role in promoting both ductal and lobular cancer proliferation [11,12]. However, some studies have shown that there is an association between the use of antipsychotic drugs that increase prolactin levels and an increased risk of breast cancer in women, although the causal relationship has not been established [34].

Antipsychotics with D2 receptor blockade and associated prolactin elevation properties have been implicated in increasing breast cancer risk [20,21]. Antipsychotic drugs that induce hyperprolactinemia, such as risperidone and pimavanserin, but not aripiprazole, may increase the risk of breast cancer by activating the JAK-STAT5 signaling pathway in precancerous lesions [35]. This effect may further explain the potential protective role of aripiprazole against breast cancer in patients with schizophrenia. Studies have found that traditional antipsychotic drugs can increase the risk of breast cancer, especially phyllodes tumors, and this has been associated with elevated prolactin levels [21]. These findings highlight the differences in the impact on prolactin levels among treatment options and suggest a potential advantage of aripiprazole in this regard.

Although hyperprolactinemia is common among schizophrenia patients, elevated prolactin levels can often be asymptomatic. Reported consequences include amenorrhea, galactorrhea, sexual dysfunction, infertility, and osteoporosis due to decreased estrogen levels [36].Consequently, some international guidelines recommend correcting hyperprolactinemia in patients treated with antipsychotics [37]. Therapeutic strategies include switching to prolactin-sparing antipsychotics, adding dopamine agonists, or incorporating D2 partial agonists like aripiprazole [19,38]. Among these strategies, adding aripiprazole has more evidence and was recommended as the first option to lower prolactin in patient with schizophrenia in a meta-analysis study [38].

Our study suggests that aripiprazole, recommended for treating antipsychotic-induced hyperprolactinemia [38] may mitigate breast cancer risk among schizophrenia patients [21]. While the association between antipsychotics and hyperprolactinemia is well-established, the evidence regarding prolactin levels and breast cancer risk is still inconclusive [38].Given the inability to directly measure prolactin concentrations in our study, further research is warranted to explore the effects of aripiprazole on prolactin levels and breast cancer risk. In our study, patients in the aripiprazole group exhibited more outpatient visits and admissions for schizophrenia. Furthermore, they demonstrated a higher prevalence of extrapyramidal symptoms and received electroconvulsive therapy more frequently (Table 2). These findings suggest that patients in the aripiprazole group may have more severe illness, leading psychiatrists to prescribe aripiprazole for refractory symptoms or to mitigate side effects like hyperprolactinemia. Despite potential increased illness severity and greater antipsychotic exposure, the aripiprazole group exhibited a reduced risk of breast cancer, suggesting a potential protective effect of aripiprazole in schizophrenia patients (Table 3).

Moreover, several studies suggest that aripiprazole may possess anticancer properties. Kim et al. showed that aripiprazole induced apoptosis and suppressed the migration of glioma cells by targeting Src in *in vitro* and *in vivo* anti-cancer activities. Src, one of several oncogenic tyrosine kinases, is activated in many types of cancer to influence survival and metastasis [39]. Aripiprazole reduced the kinase activity of Src and may have antitumor effects. Suzuki et al. demonstrated that aripiprazole inhibited the growth of serum-cultured cancer cells and cancer stem cells at non-toxic concentrations [40]. Other studies reported growth inhibition effects of aripiprazole against various cancer cell lines, including glioma, colon carcinoma, and gastric cancer [39]. Although the exact mechanisms remain unclear, in vitro studies have shown aripiprazole’s activity against breast cancer cells, inhibiting cell cycle progression and enhancing apoptosis [41]. Further investigation is warranted to elucidate aripiprazole’s anti-cancer properties, particularly its effects on breast cancer cells.

Our study has compared the psychiatric medication use between the aripiprazole group and control group. Although some studies have indicated that the use of antidepressants and benzodiazepine may increase the risk of breast cancer [42,43], the relationship between breast cancer and the use of antidepressants and benzodiazepine remains inconclusive [44]. In present study, the aripiprazole group have more psychiatric comorbidity (Table 1) and have higher cumulative dosages of antipsychotics (2,660.71 vs2,186.21, p < 0.0001), antidepressants (1,000.22vs665.73, p < 0.0001),anxiolytics (9,585.81 vs 6,596.72, p < 0.01), hypnotics (51,416 vs 39,162, p < 0.05) and still has lower prevalence rate of breast cancer compared with the control group.

Hormone replacement therapy beyond the first few years after menopause is not safe and may increase the risk of breast cancer [45]. The dramatically lowered use of menopausal hormone in the developed country has been credited with following decline in postmenopausal breast cancer incidence [46]. Women at risk of breast cancer should avoid birth control pills and hormone replacement therapy [45]. In our study, the aripiprazole group has higher cumulative dosages of hormone (175.70 vs 109.90, p < 0.0001) and still exhibited a reduced risk of breast cancer compared with the control group.

Our study shows that comorbid with alcohol using disorder may increase the risk of breast cancer in patient with schizophrenia (aHR: 1.75 95%CI 1.11–2.76)(Table 3). Studies showed approximately 50% of patients with schizophrenia have a co-occurring substance use disorder, mainly alcohol [47], they are prone to unhealthy eating habits and drink more alcohol than sex-and-age-matched peers [48,49]. Alcohol, as little as one drink per day may increase the risk of breast cancer [50]. The effects of alcohol may be mediated by the direct carcinogenic effects of metabolites such as acetaldehyde or oxygen radicals. Other hypothesized mechanisms include increased solubility of carcinogens, interference with foliate or estrogen metabolism, or nutrient deficiencies associated with high alcohol use [51]. Patients with alcohol use disorder are widely underdiagnosis and undertreatment [52]. Psychiatrist should actively inquire alcohol use issue with patients with schizophrenia and give enough information about the treatment for alcohol use disorder, especially in patients with high risk of breast cancer.

Another important factor increased the prevalence rate of breast cancer among patients with schizophrenia is obesity and metabolic syndrome. Patients with schizophrenia lead a relatively sedentary life, indulge in unhealthy eating habits and may induce obesity [48]. Furthermore, the introduce of atypical antipsychotics have reduced the neurological side effects of antipsychotics, but the metabolic side effects such as DM, obesity, metabolic syndrome dramatically increase among patients with schizophrenia [53]. Obesity and DM are important risk factors for breast cancer [54]. Aripiprazole has been classified in low body weight increasing agent among atypical antipsychotics [55]. In our study, the aripiprazole group has more obesity, hypertension, hyperlipidemia, and cardiovascular disease. (Table 1) and exhibited a reduced risk of breast cancer. It is possible that clinician tend to choose aripiprazole to treat schizophrenic patients prone to metabolic syndrome to prevent further aggravation of syndrome. To reduce the risk of breast cancer, clinicians should try to prevent metabolic side effects in patients with schizophrenia by judicious prescription of antipsychotics and encourage healthy lifestyle such as exercise and diet programs.

In addition to aripiprazole, other antipsychotics with partial dopamine agonist properties, such as brexpiprazole and cariprazine, have shown fewer prolactin-elevating side effects. However, these medications are relatively new to Taiwan, and further clinical data are required for analysis. Future studies should evaluate other partial dopamine agonists’ efficacy in preventing breast cancer.

Strengths of our study include its nationwide, population-based design, minimizing selection bias by tracing all schizophrenia cases in Taiwan during the study period. Additionally, the large sample size provided adequate statistical power to detect subtle differences between cohorts. However, several limitations warrant consideration. Firstly, data on suspected breast cancer risk factors, including smoking, dietary habits, and body mass index, were unavailable, potentially introducing bias. Secondly, family history of breast cancer, a recognized risk factor, could not be assessed. Thirdly, patient compliance with prescribed psychotropic therapy could not be determined. Fourthly, adjustments for physical exercise, lifestyle factors, and prolactin concentrations were not feasible. Lastly, the observational nature of our study may have underestimated the relative correlation due to unmeasured confounding factors. Further studies should address these limitations by evaluating breast cancer prevalence with and without aripiprazole use, ideally adjusting for prolactin concentrations.

## Conclusions

Summarized, our study indicates that aripiprazole may confer protective effects against breast cancer in women with schizophrenia. Considering the higher prevalence of breast cancer in women with schizophrenia compared to the general population, monitoring prolactin concentrations when prescribing antipsychotics and incorporating aripiprazole to lower prolactin levels in patients with hyperprolactinemia should be considered, particularly in those with a family history of breast cancer. Mental health professionals should also be mindful that breast cancer in schizophrenia patients is often under-diagnosed and under-treated. Thus, it is crucial for psychiatrists to educate patients and their families about maintaining a healthy lifestyle and undergoing appropriate cancer screening procedures.
